# Multi-Perspective Analysis of Building Orientation Effects on Microstructure, Mechanical and Surface Properties of SLM Ti6Al4V with Specific Geometry

**DOI:** 10.3390/ma14164392

**Published:** 2021-08-05

**Authors:** Wentong Cai, Qinghua Song, Hansong Ji, Munish Kumar Gupta

**Affiliations:** 1 Key Laboratory of High Efficiency and Clean Mechanical Manufacture, Ministry of Education, School of Mechanical Engineering, Shandong University, Jinan 250061, China; efficientc@126.com (W.C.); hansgji@163.com (H.J.); munishguptanit@gmail.com (M.K.G.); 2 National Demonstration Center for Experimental Mechanical Engineering Education, Shandong University, Jinan 250061, China; 3 Faculty of Mechanical Engineering, Opole University of Technology, 45-758 Opole, Poland

**Keywords:** building orientation, selective laser melting, Ti6Al4V, microstructure, mechanical properties

## Abstract

Building orientation is important in selective laser melting (SLM) processes. Current studies only focus on the horizontal and vertical building orientations without considering different modes of horizontal orientations. In fact, for horizontal orientation, different surfaces of the sample that contact the substrate will affect the heat transfer mode and efficiency, and in turn affect the microstructure and material properties. In this paper, the effect of two modes of horizontal building orientations on microstructure, mechanical and surface properties of SLM Ti6Al4V was studied. Current research about building orientation is deficient because the geometry of samples or test surfaces are not strictly defined, which seriously influences the results due to their different heat transfer efficiency and mode. Therefore, the geometry of the samples and test surfaces were clearly defined, and its necessity was proved in this study. To achieve the research goal, three test samples were prepared: sample SLM-PB-S with the building orientation parallel to the substrate and the shorter side *L*1 contacts it, sample SLM-PB-L with the building orientation parallel to the substrate and the longer side *L*2 contacts it and sample SLM-VB with the building orientation vertical to the substrate. Subsequently, the microstructure, grain information, densification, residual stress, micro-hardness, tensile properties and surface topography of different samples were analyzed and compared. In the results, SLM-PB-S exhibited denser microstructure and better mechanical properties than SLM-PB-L, including smaller grain size, stronger texture, higher density, micro-hardness, tensile strength, plasticity and better surface quality. It originates from a higher cooling rate and shorter scanning time between layers during SLM-PB-S fabrication, leading to finer grains, lower porosity and better interlayer metallurgical bonding, thus resulting in better material properties. This study can provide a reference to select the proper building orientation in SLM.

## 1. Introduction

Ti6Al4V is a typical α + β type titanium alloy [1]. It has the characteristics of high specific strength, good corrosion resistance, excellent biocompatibility and sound comprehensive mechanical properties. Therefore, it has been widely used in aerospace, ship and medical equipment and other fields [2,3,4]. Additive manufacturing (AM) technology encompasses multiple disciplines of physics, machinery, materials, etc. Compared with milling, turning, grinding and other traditional subtractive manufacturing technologies, additive manufacturing has a great advantage in rapid prototyping, independent design and accessibility of complex parts [5,6]. However, poor surface morphology, surface roughness, low mechanical properties and dimensional accuracy are the main disadvantages of AM [7,8,9]. Therefore, the study on AM materials has aroused the interest of many researchers who try to improve the properties of the materials.

Among the various AM processes, SLM is an advanced and reliable technology that is widely used to fabricate metal parts and there is almost no material loss in the process [10,11,12,13]. In the past two decades, the technology of SLM has been studied extensively. To improve the microstructure and mechanical properties of the parts made by SLM, there are currently two main approaches. One is to rely on post-processing procedures, and the other is to optimize the SLM process parameters. For the first approach, Han et al. [14] conducted post-processing research on selective laser melted AlSi10Mg by two methods (550 °C for 2 h, followed by cooling and laser surface remelting), respectively. It was found that the tensile strength of the workpiece decreased after heat treatment, while the ductility increased due to the growth of crystal grains and the release of residual stress. The laser surface remelting process improved the surface roughness of the sample and in the meantime, increased the microhardness of the sample by 19.5%. Yan et al. [15] investigated the microstructure and tensile strength of Ti6Al4V ELI samples made by SLM after heat treatment under vacuum and hot isostatic pressing conditions. They found that hot isostatic pressing and vacuum heat treatment can significantly reduce the number of pores and cracks inside the sample, reduce the strength and improve the ductility and fatigue properties of the material. Compared with the sample heat-treated under vacuum, the ductility and fatigue resistance of the sample heat-treated under hot isostatic pressing improves greatly. K. Karamia et al. [16] investigated the effects of optimal way of combination for sand blasting, thermal isostatic pressure and chemical etching on the mechanical and fatigue properties of Ti6Al4V and found that the combination of sand blasting and thermal isostatic pressure and chemical etching improved the fatigue behavior of Ti6Al4V the most. Although the above conventional post-treatment methods have a certain effect on the improvement of mechanical properties of parts, they are insignificant compared to the microstructural optimization and the improvement of mechanical properties for materials caused by changing SLM process parameters [17] and are not direct and economical. Therefore, more researchers have focused their research on the optimization of process parameters in the SLM. Sun et al. [18] studied the impact of the laser parameters on porosity, surface morphology and tensile strength of Ti6Al4V samples manufactured by SLM. The results showed that the relative density of the formed sample could reach 99% with the decreasing of scanning speed and the increasing of laser power under the condition of relatively high laser power (higher than 175 W). Lee et al. [19] found that if the laser power is too high or too low, due to the powder spheroidization or melting deficiency, defects such as holes would appear in the alloy and cause deformation or cracks in the formed parts. Xia et al. [20] examined the effect of hatch spacing on thermodynamics and resultant surface quality. They found that proper hatch spacing could guarantee reasonable temperature, which is beneficial to the formation of a smooth surface of part. When the hatch spacing was 60 μm, the average surface roughness was 2.23 μm. Qiu et al. [21] studied the effect of powder thickness on surface structure and porosity of Ti6Al4V fabricated by SLM and considered the melt flow behavior. The observation showed that the powder thickness imposed a greater impact on the forming quality than the scanning speed and the laser power did. Surface roughness and porosity of the alloy increased when the thickness was larger than 0.04 mm. Through finite element simulation, Song et al. [22] found that the residual stress of Ti6Al4V alloy was the smallest when the laser rotation angle was 15°. According to literature review, parameters related to the AM process such as scanning speed, laser power, hatch spacing and scanning strategy have been widely studied.

**Figure 1:**
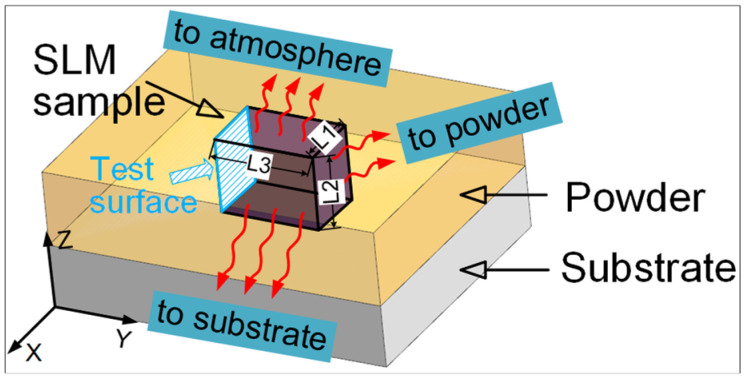
Schematic diagram of heat transfer during sample manufacturing (*L*3 > *L*2 > *L*1).

In recent years, some researchers have begun to study a relatively novel parameter named building orientation. In 2018, He et al. [23] studied the microstructure and tensile properties of different sides of SLM Ti6Al4V, but it is worth noting that the geometry of different sides in this study were not the same. In fact, different geometries will affect the forming process of parts, leading to different microstructure and mechanical properties. This is because the different geometries correspond to different heat transfer modes and efficiency, which in turn affects the grain size and crystallization process. In 2019, Ren et al. [24] set five gradients from 0–90° to study the effect of building orientation on the microstructure and mechanical properties of SLM Ti6Al4V, but there was a significant difference between the 0° sample and other samples in geometry, which would have a great impact on the reliability of the data. In 2020, Chang et al. [25] and Xie et al. [17] studied the effect of building orientation on the microstructure and mechanical properties of SLM Ti6Al4V, but they considered the horizontal and vertical directions only. In fact, for the horizontal building orientation, different surfaces of the sample in contact with the substrate will affect the heat transfer mode and efficiency during SLM, as shown in Figure 1. It will affect the crystallization process and interlayer metallurgical bonding of crystal grains. Different microstructures and mechanical properties will be displayed. At the same time, the surface properties of the test surface of the formed sample will be greatly affected. However, the effect of different horizontal building orientations on microstructure, mechanical and surface properties of SLM Ti6Al4V was rarely studied before. In addition, previous studies analyzed the influence mechanism of building orientation on the microstructure and mechanical properties from several perspectives such as SEM morphology and tensile properties, which were not comprehensive. Consequently, the influence mechanism of building orientation on mechanical properties is not clear. Moreover, there are few studies on microhardness and residual stress, which are also important mechanical properties of materials. In addition, the residual stress has significant influence on the tensile and fracture behaviors of the material.

This paper aims to solve the three problems: different geometries for samples affect the research results, which is not recommended; previous research perspectives are incomplete; and the effect of different horizontal building orientations has rarely been studied before. For horizontal building orientation, the influence of two different contact modes with the substrate on the microstructure, mechanical and surface properties of the SLM samples was studied. The sample printed in the vertical building orientation was used as a reference. The SLM Ti6Al4V samples and the test surfaces were limited to have the same geometry in the research process, and its necessity was proved by comparisons with the existing results in the literature. Multiple perspectives were used to explore the influence mechanism of building orientation on microstructure, mechanical and surface properties, including microtopography, texture, density, micro-hardness, tensile properties, residual stress and surface morphology. A more reasonable mapping relationship among process (orientation), microstructure and performance for SLM Ti6Al4V was established.

## 2. Materials and Methods

### 2.1. Sample Preparation

**Figure 2:**
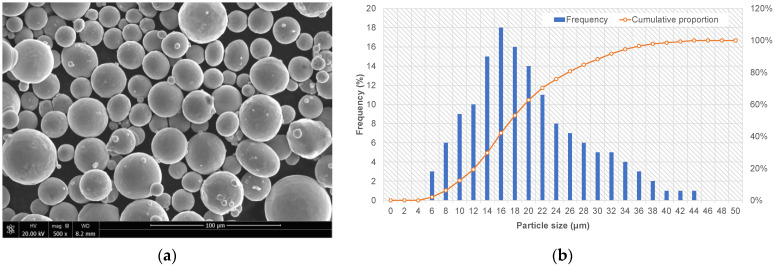
The powder information. (**a**) The microscopic morphology; (**b**) particle size distribution diagram of the Ti6Al4V powder used in the SLM process.

**Figure 3:**
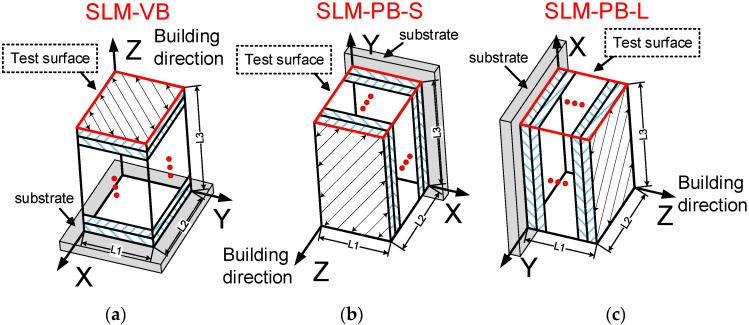
Samples fabricated by SLM in different building orientations. (**a**) SLM-VB; (**b**) SLM-PB-S; (**c**) SLM-PB-L.

Ti6Al4V powder used in the SLM process is provided by the “3D SYSTEMS” company, Rock hill, SC, USA. The chemical components are shown in Table 1. Figure 2 shows the morphology and size information of the powder used in the SLM process. It can be seen from Figure 2a that the morphology of powder particles is spherical or subspherical, and the surface is smooth and intact without obvious defects. Therefore, it is qualified as the raw material for the SLM manufacturing process. Figure 2b shows the particle size distribution of powder with a diameter ranging between 6 μm and 44 μm, which meets the requirements of the SLM process. The manufacturing machine equipped with fiber laser 80 μm in diameter is ProX DMP 320, acquired from “3D SYSTEMS” company in the United States. The manufacturing process was conducted in an argon-filled environment and the concentration of oxygen gas is lower than 5 ppm, which prevented the metal powder from being oxidized during melting. Three samples (SLM-VB, SLM-PB-S and SLM-PB-L) with a size of 5 mm × 6 mm × 8 mm were used to photograph the microscopic appearance and test the mechanical properties. The samples were printed in different building orientations with a size of 5 mm for *L*1, 6 mm for *L*2 and 8 mm for *L*3 (Figure 3), respectively. It is worth noting that sample SLM-VB was with the building orientation vertical to the substrate, sample SLM-PB-S was with the building orientation parallel to the substrate and the short side *L*1 of sample coming into contact with it and sample SLM-PB-L was with the building orientation parallel to the substrate and the long side *L*2 coming into contact with it. Subsequently, the specimens for tensile test were prepared. The rotation angle between layers was 72° during scanning. The specific parameters in SLM process are shown in Table 2. Among them, volume energy density (E) is 58.94 J/mm^3^, obtained by Equation (1) [26,27]:

(1) $$E=\frac{P}{V\cdot T\cdot h}$$

where *E* is volume energy density (J/mm^3^), *P* is laser power (W), *V* is scan speed (mm/s), *T* is hatch space and *h* is layer thickness (mm).

### 2.2. Microstructure Observation

Optical microscope (OM) (Feng Zhi, Jinan, China) was used to evaluate the microstructure of SLM Ti6Al4V samples. Firstly, the samples were polished with different sizes of emery papers (mesh size 100–3000) followed with diamond paste and fine size alumina powder. Then, Kroll’s reagent was used as the etchant.

It is worth noting that the grain size and orientation of SLM Ti6Al4V samples were observed with the NordlyMax3 electron backscatter diffraction (EBSD) system equipped in the field emission scanning electron microscope (FESEM) JSM-7800 (Manufacturer: JEOL, Tokyo, Japan). Magnification 1000×, accelerating voltage 20 kV, working distance 20.0 mm and sample tilt angle 70° were set in the system. The step size was 0.1 μm and the mapping area was 60 μm × 40 μm. Before observation, the emery papers with mesh size 240–2500 were used to grind the surface of the samples successively. Then, argon ion polishing was used, and the polishing process required the use of liquid nitrogen to create an environment of −35 °C. Commercial ion polishing equipment type was ArBlade5000 and ion beam voltage was 3.5 kV.

### 2.3. Measurement of Mechanical Properties, Residual Stresses and Surface Topography

Archimedes’ principle was selected to measure the density of SLM samples [4]. The specific equation is as follows:

(2) ρRe=ρρTh×100%

where *ρ*_Re_, *ρ* and *ρ*_Th_ are the relative density, average density value and theoretical density of the material (4.43 g/cm^3^), respectively.

The microhardness test was conducted on a Future-tech (MH-6) machine (FUTURE-TECH, Tokyo, Japan) according to ASTM E384-17 where the load was 0.5 kg and dwell time was 8 s. We used the X-ray residual stress measurement system μ-X360n (Pulstec, Hamamatsu, Japan) to measure the residual stress of samples. The specimens for tensile testing were prepared in accordance with the GB/T 228.1-2010 standard, and the experiment was conducted at a rate of 0.01 mm/s at room temperature. The model of the machine in this test was Instron 5582 (Instron, Boston, MA, USA). The surface topography of the samples was analyzed using laser scanning confocal microscope (Keyence, VK-X200 series, Osaka, Japan). All of the measurements were conducted at five different locations and each final value of micro-hardness, residual stress and surface roughness was the average of the five tests.

## 3. Results and Discussion

### 3.1. Microstructure

**Figure 4:**
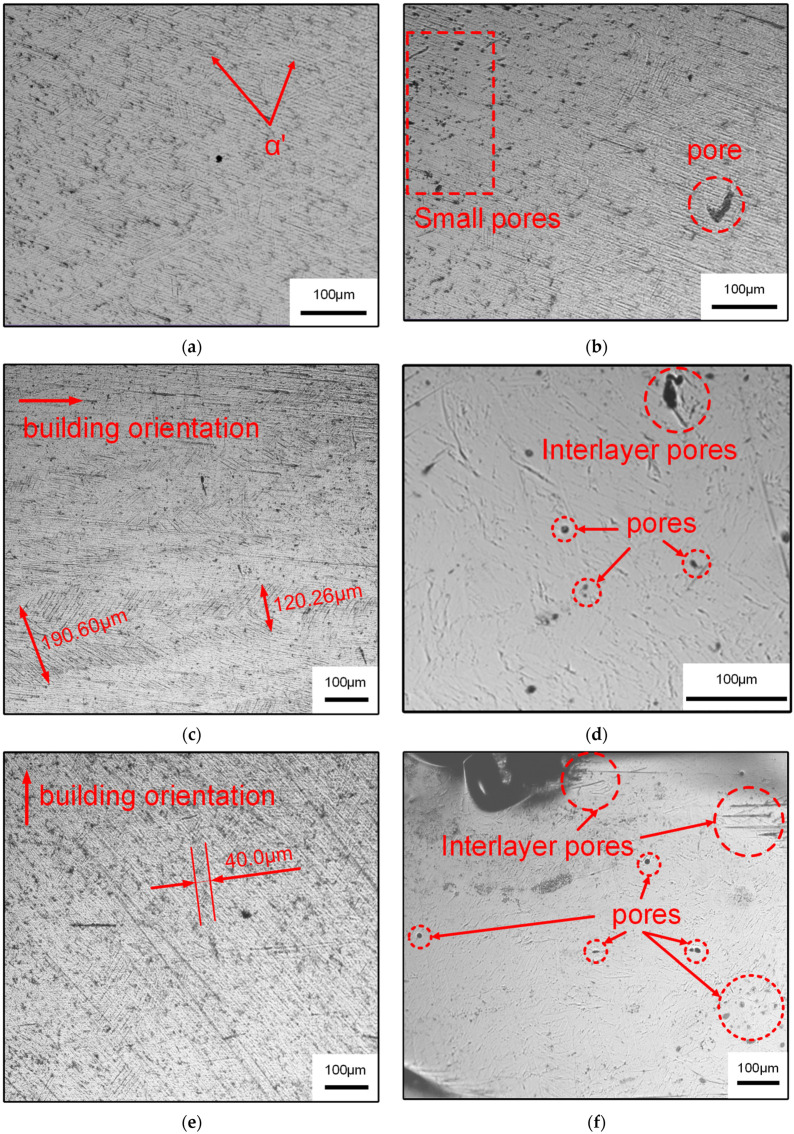
OM micrographs of different samples. (**a**,**b**) SLM-VB; (**c**,**d**) SLM-PB-S; (**e**,**f**) SLM-PB-L.

Ti6Al4V is a typical alloy with dual phase (α + β), and its performance is closely related to the microstructural morphology [5]. The close-packed hexagonal α phase and the body-centered cubic β phase constitute the basic phase of Ti6Al4V alloy [28]. The initial microstructure of Ti6Al4V titanium alloy mainly depends on the cooling rate during the forming process. The higher the cooling rate, the finer the structure. Because the cooling rate of the SLM process was as high as 10^5^–10^6^ K·s^−1^ [29], the microstructure formed was fine, mainly in the form of dendrite. Figure 4 shows the microstructure of Ti6Al4V formed by SLM under an optical microscope. It can be seen that a large number of α’ acicular martensite grains were formed in the original columnar grains, as shown in Figure 4a. Due to the fact that the β phase did not have the time to convert to α phase during the rapid solidification of the alloy, tiny acicular martensite was obtained [6,7]. It can be seen in the test surfaces of SLM-PB-S and SLM-PB-L that primitive columnar crystals were found to grow along the building orientation. This is because the powder melted and formed Ti6Al4V melt and nucleated to grow with the substrate as the core in the SLM process when the laser acted on the first layer of Ti6Al4V powder. In this process, the cooling rate and temperature gradient were large, and the grain growth direction was consistent with the temperature gradient (perpendicular to the direction of the substrate). As the subsequent layering melted and solidified, the melt in the molten pool of the next layer nucleated to grow as the core of the base section of the previous layer, and the heat is mainly conducted along the molten pool to the forming direction. Finally, the epitaxially grown β columnar crystal was formed along the direction of heat conduction. A large number of acicular α’ grains were distributed in parallel in the columnar crystal, which was consistent with the research results of Xie et al. [17]. It was found by further observation that most of the martensite α’ nucleated at the primary β grain boundary. The width of the primary β phase in SLM-PB-S was 120.26–190.60 μm, as shown in Figure 4c. It is similar to the results in the literature [23]. However, the width of the primary β phase in SLM-PB-L was only 40.0 μm, as shown in Figure 4e, which is a unique conclusion compared to existing literature. In addition, the porosity of the material also changed gradually. Figure 4b shows that there are small pores on the plane of SLM-VB and no large pores are observed. SLM-PB-S has larger interlayer pores, as shown in Figure 4d. The number and area of interlayer pores in SLM-PB-L are significantly larger than SLM-PB-S, as shown in Figure 4f. It will have a certain impact on the mechanical properties of the materials.

### 3.2. EBSD Analysis

**Figure 5:**
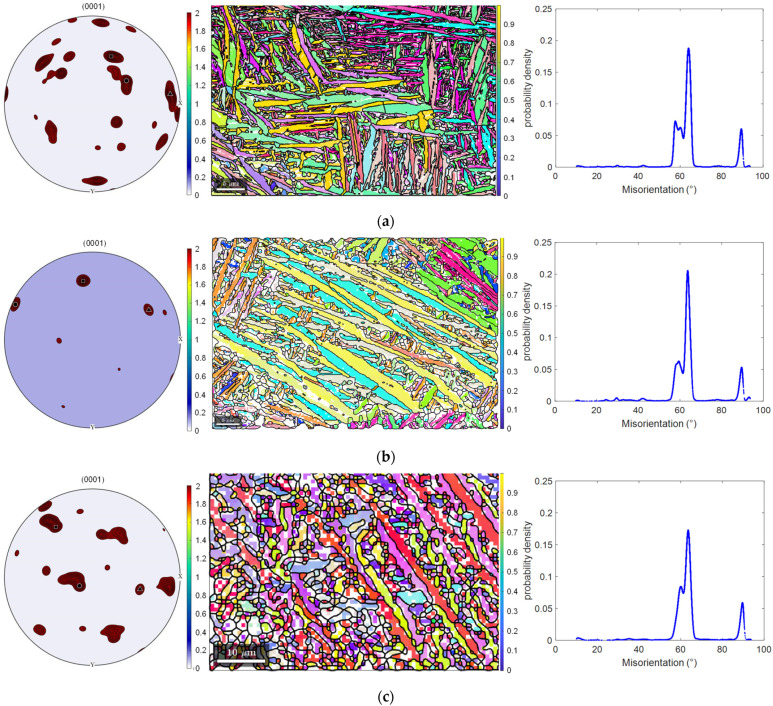
Pole figures, orientation distribution figures (with lattice) and misorientation distribution figures. (**a**) SLM-VB; (**b**) SLM-PB-S; (**c**) SLM-PB-L.

The orientation and the geometry information of grains impose a great impact on the mechanical properties of Ti6Al4V alloys. Therefore, it is necessary to conduct an additional study of grain size, grain shape and orientation of the test surface of SLM-VB, SLM-PB-S and SLM-PB-L. Different samples were analyzed by EBSD data such as pole figures (PF), orientation distribution data and misorientation distribution figures. The grains’ information data were calculated by Mtex-toolbox in Matlab. Considering the crystal structure, lattice constants or the orientation relationship with the parent phase, α’ phase and α phase are almost the same. Hence, the content of this part is uniformly described as α phase. Table 3 shows the partial grain geometry information (mean area, aspect ratio) of different samples. There are three columns of pictures in Figure 5. The first column on the left shows the crystal textures of the test surfaces by pole figures. The second column shows the grain morphologies and grain orientations, and different colors present diverse orientations. The third column shows the distribution of grain misorientations. It can be seen that the grain misorientations are mainly distributed in 60–70° for the three samples, and SLM-PB-S has the highest probability density, leading to the most dislocation accumulation. Further, it may result in good mechanical properties that will be discussed in later content. Figure 5a–c correspond to samples SLM-VB, SLM-PB-S and SLM-PB-L. Moreover, the volume fraction for preferred orientation (VFPO) of grains and grain size (Table 4) were calculated in Matlab software. According to the calculation, the volume fraction gradually reduced in the order of SLM-PB-S, SLM-VB and SLM-PB-L. However, the grain size increased in the order of SLM-VB, SLM-PB-S and SLM-PB-L (0.7566 μm < 1.3032 μm < 1.9453 μm), as shown in Table 4. The smaller grain size was caused by a higher cooling rate in the SLM process. At the higher cooling rate, the grains did not have enough time to grow. Further, the test surface of SLM-VB was at the top during the SLM process, and it had big area exposed to the atmosphere and large thermal gradient, which led to the high cooling rate. SLM-PB-S and SLM-PB-L, meanwhile, were not exposed to the atmosphere and were blocked by powder in the SLM process. The powder reduced the rate of heat conduction, and then the heat accumulation effect was obvious. Therefore, the samples SLM-PB-S and SLM-PB-L had slower cooling rate and lager grain size. Moreover, these two samples formed by stacking cladding powder layer by layer. When the next layer was scanned, heat transferred to the last layer of powder. The process reduced the thermal gradient of the previous layer of powder cooling and extended the time for grains to growth. Therefore, SLM-PB-S and SLM-PB-L had larger grain area and smaller aspect ratio (Table 3).

From another point of view, Yang et al. [30] found that in the SLM process, the microstructure of the material (grain size and phase composition, etc.) was affected by the distance from the molten pool to the substrate, and that greater distance produced greater thermal gradient. For SLM-PB-S and SLM-PB-L, the distance *L*2 (Figure 3) from SLM-PB-S to substrate was greater than *L*1 from SLM-PB-L to substrate, so SLM-PB-S had a larger temperature gradient than SLM-PB-L during the melting and solidification process, which led to a higher cooling rate. Therefore, the grain size of SLM-PB-S was smaller than that of SLM-PB-L (Table 4). The high cooling rate in the SLM process led to the high proportion of α’ phase. Rapid heat conduction caused thermal undercooling and dynamic undercooling in the melt pool. Then, columnar β phase transformed to α’ phase. According to the Burgers relation, the β phase transformation to α’ phase abides by Equations (3) and (4) [31]:

(3) $${\left(100\right)}_{\beta }↔{\left(0001\right)}_{\alpha }$$

(4) $${\langle 1\bar{1}1\rangle }_{\beta }↔{\langle 11\bar{2}0\rangle }_{\alpha }$$

All the grains in SLM-VB, SLM-PB-S and SLM-PB-L surface were micron in size. The shape of grains in SLM-VB, SLM-PB-S and SLM-PB-L followed the rule that the grain shape changed from fine columnar to coarse columnar. We can draw the conclusion from the mean area and mean aspect ratio in Table 3. The grain morphology information also demonstrated the same rule in the second column of Figure 5. Table 3 shows that the mean grain areas are 0.3451 μm^2^, 1.0761 μm^2^ and 2.3986 μm^2^ and the mean grain aspect ratios (length/width) are 3.4906, 3.1953 and 2.6612, respectively. The max grain aspect ratios (length/width) are 18.9547, 17.2892 and 8.4321 for SLM-VB, SLM-PB-S and SLM-PB-L, respectively. The fine grains may lead to good mechanical properties, which we discuss later in this paper. The preferred orientations of grains were different in different surfaces and we can obtain them from volume fractions (Table 4) and pole figures (the first column in Figure 5). Table 4 shows that the volume fractions of the preferred orientations were 21.4714%, 33.1181% and 20.2455% for SLM-VB, SLM-PB-S and SLM-PB-L, respectively. The pole figures more vividly show the strength of the texture for different samples. It was obvious that SLM-PB-S had the strongest texture and SLM-VB and SLM-PB-L were slightly weaker, which can also be seen from the grain orientation information in the second column of Figure 5. SLM-PB-L had the weakest texture of the samples. An interesting phenomenon was found, that the growth of grains was based on the preferred orientations from Figure 5. Most of the (1000) lattice planes were close to the extension direction perpendicular to the grains. This is because the surface energy of (1000) is the largest and the growth rate is the fastest. Table 5 shows two-phase ratios of SLM-VB, SLM-PB-S and SLM-PB-L, which were calculated by Mtex tool box in Matlab. By analysis, α phase accounted for 99.78% and β phase accounted for 0.22% in SLM-VB, while in SLM-PB-S, α phase accounted for 99.43% and β phase accounted for 0.57%. The volume fractions of dual phases are closely related to the cooling rate in the SLM process. A higher cooling rate tends to result in more transition from β phase to α phase. The data size relationship of biphasic ratio (β/α) between SLM-VB and SLM-PB-S in this paper and samples in the literature [17] is the same. The relative change in the biphasic ratio (β/α) of these two samples is 61.19%, which is quite different from 35.54% in the literature [17]. This may be due to the fact that SLM-2 is smaller in size compared to SLM-1 in the literature [17], resulting in a higher conversion rate from β phase to α phase. Therefore, it is necessary to limit the sample size and test surface size while studying the impact of building orientation on microstructure. In SLM-PB-L, α phase accounts for 99.46% and β phase 0.54%. The volume fraction of α phase and β phase and their respective properties have important effects on the final mechanical properties of the samples. Compared with α phase, β phase has higher plasticity but lower strength and hardness. Therefore, the proportion of α and β phases will have an important effect on the mechanical properties of materials, discussed later in this paper.

### 3.3. Densification

**Figure 6:**
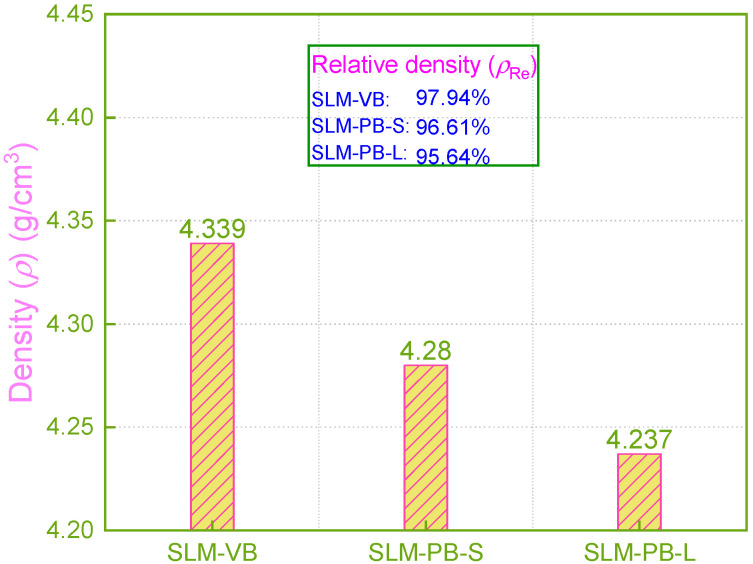
Density of different samples.

Density is an important index to measure the manufacturing quality of additive manufactured parts [32]. Generally, due to its layered and superimposed manufacturing process characteristics, the SLM manufacturing method has a high degree of freedom in processing, which can be used in the manufacturing of complex structural parts that are hard to prepare by conventional material reduction technology [33]. It greatly improves the production efficiency. However, in the SLM process, with high laser heat input and high temperature gradient, the melting and solidification process of powder will be completed in a very short time, and the gas in the melt cannot be completely split in a short time, causing pores in the tissue and affecting the density of the formed samples [32]. The density of the samples was measured by Archimedes’ principle. The measurement result showed that the relative density of the samples decreased in the order of SLM-VB, SLM-PB-S and SLM-PB-L, and the values were 97.94%, 96.61% and 95.64%, respectively, as shown in Figure 6. The building orientation of the samples was the main reason for the density difference. SLM-VB was formed by laser scanning, with relatively sufficient powder melting, it had less unmelted powder on the surface and low porosity. However, SLM-PB-S and SLM-PB-L were formed by powder melting and stacking forming, so large pores easily formed between layers with large thermal gradient value. In addition, because the latter surface needed to be rotated by 72° on the basis of the previous surface during the scanning process, the thermal gradient values of different areas of the forming surface during the interlayer stacking process were greatly different. It caused the melt to flow unsteadily and the powder to melt inadequately. Eventually, many pores were formed in the samples. The difference in porosity between SLM-PB-S and SLM-PB-L was mainly because different surfaces contacted the substrate. SLM-PB-S was stacked layer by layer with an area of *L*1 × *L*3 mm^2^, while SLM-PB-L was stacked layer by layer with an area of *L*2 × *L*3 mm^2^. Therefore, the stacking area of SLM-PB-S was smaller, which resulted in shorter single-layer scanning time, and the heat was transferred from the next layer to the previous layer more efficiently. Further, the powder melted more fully, the melt flowed more steadily and spread more evenly, the porosity was lower and the relative density value was higher.

In this section, the data of SLM-VB and SLM-PB-S have the same size rule with 0°and 90° samples in the literature [24]. It is worth noting that the difference between the two values in this paper is 1.33%, while the difference in the literature [24] is 0.43%. This demonstrates that it is necessary to limit the geometry of the samples fabricated under different building orientations.

### 3.4. Residual Stress

The residual stress analysis of SLM Ti6Al4V fabricated under three different building orientations is novel. Generally, residual stress is harmful in that it causes material deformation and cracks, which adversely affect the structural integrity and service performance of the material [34,35,36]. Due to the high temperature gradient and rapid melting and cooling process in the SLM process, it is easy to produce uneven plastic deformation, resulting in residual stress [2,34]. Experimental residual stress results of SLM samples are shown in Table 6. The calculation principle is the Von Mises principal stress model:

(5) $$\sigma_{eq}=\sqrt{\frac{1}{2}[{\left(\sigma_{a}-\sigma_{b}\right)}^{2}+{\left(\sigma_{b}-\sigma_{c}\right)}^{2}+{\left(\sigma_{c}-\sigma_{a}\right)}^{2}+6(\tau_{ab}^{2}+\tau_{bc}^{2}+\tau_{ca}^{2})]}$$

where *σ_eq_* is identified as equivalent stress; *σ_a_*, *σ_b_* and *σ_c_* are stress components in X, Y and Z direction in cartesian coordinates; and *τ_ab_*, *τ_bc_* and *τ_ca_* are shear stress corresponding to XY, YZ and ZX planes.

The data show that the residual stress is all tensile stress (+). It is not expected. In fact, compressive stress is beneficial to the mechanical properties of materials and prevents the initiation and propagation of cracks to a certain extent [34,37]. On the contrary, tensile stress promotes the initiation and propagation of cracks. In the AM process, residual stress is generated during the solidification of the powder after melting, and the thermal gradient and shrinkage are the main reasons for the residual stress of parts [38]. In addition, related research shows that an important factor affecting the residual stress of the workpiece is the residual heat in each layer of the part. If more residual heat remains in the part, it will have greater residual stress value [39]. The experimental results show that the residual stresses of the SLM samples are all tensile stresses, and SLM-VB > SLM-PB-S > SLM-PB-L. SLM-VB had the highest temperature gradient during the SLM process, and its distance to the substrate was significantly higher than that of SLM-PB-S and SLM-PB-L. This was a major reason why the sample SLM-VB had the largest temperature gradient. In addition, the results of sample densification (Figure 6) show that SLM-VB had the highest density value among the three samples and the smallest porosity (see Figure 4). Low porosity is detrimental to the diffusion of residual heat, so its residual heat in the part led to the greatest residual stress. Compared with SLM-PB-L, SLM-PB-S had a larger distance to the substrate and lower porosity, so it had a higher temperature gradient and more residual heat remaining in the parts. Accordingly, the residual stress value of SLM-PB-S was greater than that of SLM-PB-L.

In this section, the data size relationship between SLM-VB and SLM-PB-S is the same in this paper as in samples 0° and 90° in the literature [24]. The relative change in the residual stress of the first two samples is 35.07%, which is quite different from 48.95% in the literature [24]. This is due to the different size and shape used in 0° and 90° samples in the literature [24], which led to the difference in thermal gradient and heat transfer efficiency. Therefore, it is necessary to limit the sample geometry while studying the impact of building orientation on mechanical properties.

### 3.5. Micro-Hardness

**Figure 7:**
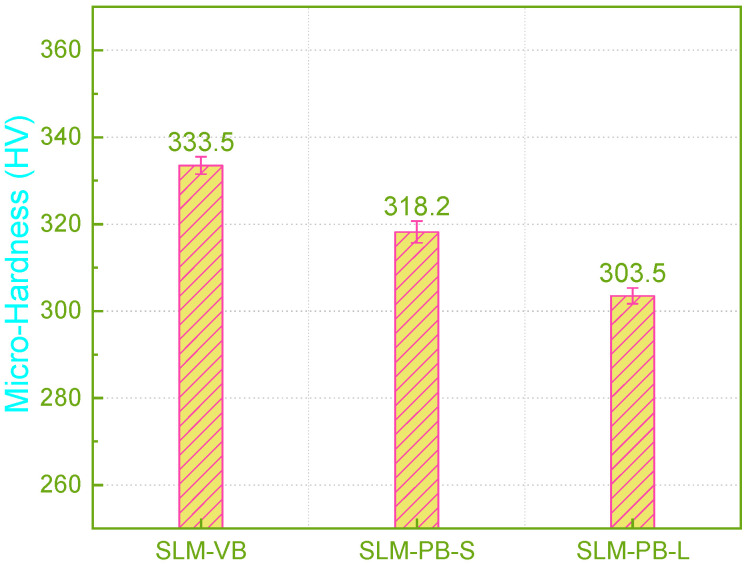
Micro-hardness values of samples.

Currently, research on the building orientation of SLM Ti6Al4V rarely involves the measurement of micro hardness, but this is an important factor of mechanical properties. In this paper, the micro-hardness of the SLM samples were measured and the result were given in Figure 7. During the forming process of SLM, there was a large temperature gradient inside the molten pool and the existence time of the molten pool was extremely short. In the process of β phase transition, the β phase did not have time for the equilibrium transformation, and directly formed the martensite α’ structure. The fine acicular martensite structure caused the formed specimens to exhibit higher hardness. By measuring the micro-hardness, it is found that the microhardness of the SLM samples decreased (333.5 HV > 318.2 HV > 303.5 HV) gradually as SLM-VB, SLM-PB-S and SLM-PB-L. According to the results of EBSD analysis, this was mainly caused by the gradual refinement of the sample grain. On the other hand, grain refinement increased the number of grain boundaries, which improved the surface resistance to indentation of the samples, and thus improved the micro-hardness. In addition, due to the difference in mechanical properties between α phase and β phase, the microhardness of samples will also be affected. Generally, the hardness and strength of α phase are higher than β phase, so the distribution of α phase and β phase also has an important influence on the microhardness of the materials. With the sample change according to SLM-VB, SLM-PB-L and SLM-PB-S, the proportion of α phase showed the following pattern as shown in Table 5: SLM-VB (99.78%) > SLM-PB-L (99.46%) > SLM-PB-S (99.43%). It can be seen clearly that SLM-PB-S and SLM-PB-L were very similar in the proportion of α phase, but the proportion of α phase in SLM-VB was significantly higher than the other two. Therefore, the microhardness of SLM-VB was significantly higher than that of SLM-PB-S and SLM-PB-L. In addition, sample density had the following size relationship (see Figure 7): SLM-VB > SLM-PB-S > SLM-PB-L. The porosity of the samples had the following relationship: SLM-VB < SLM-PB-S < SLM-PB-L (see Figure 4). The two aspects were also the main reasons for the change in the hardness of the samples’ regularity. On the other hand, due to the large temperature gradient during the forming process, the residual stress inside the formed specimens increases the hardness to a certain extent. The residual stress data of the samples are given in Table 6. This counts for the fact that SLM-VB has the largest hardness and SLM-PB-L has the smallest hardness. To sum up, smaller grain size, more grain boundaries, higher content of α phase, greater density and smaller porosity resulted in higher micro-hardness value of samples.

### 3.6. Tensile Properties

**Figure 8:**
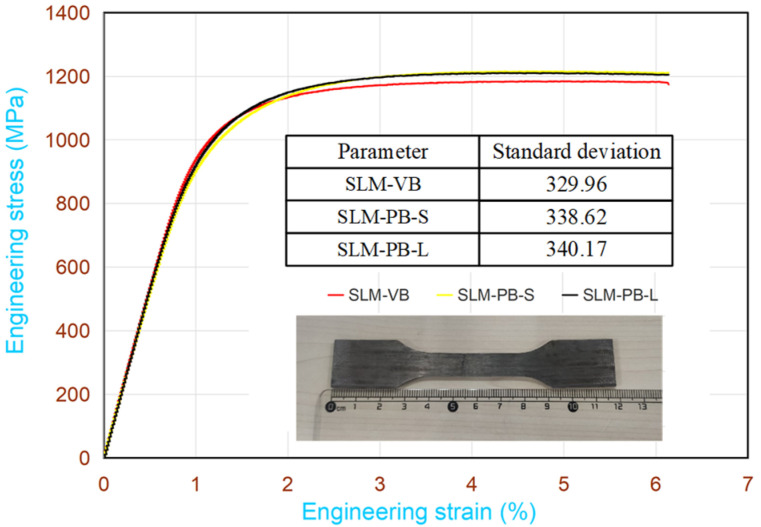
Stress-strain curves for tensile tests.

**Figure 9:**
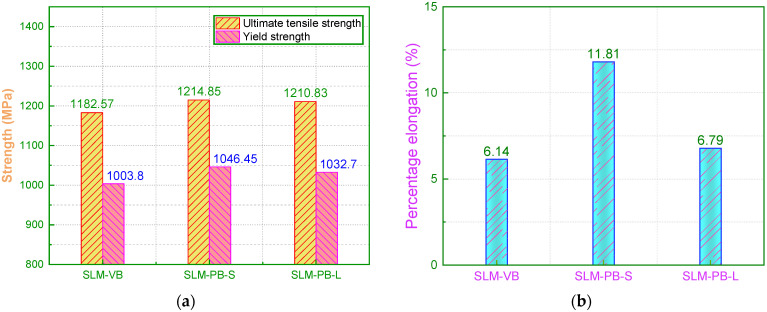
Results of tensile properties for SLM Ti6Al4V samples. (**a**) Ultimate tensile strength and yield strength data; (**b**) percentage elongation data.

To obtain the strength and plasticity of the samples, the tensile test was conducted. Figure 8 shows the engineering stress-strain curves of the three samples. The relationship among the tensile strength of three samples was as follows: SLM-PB-S > SLM-PB-L > SLM-VB. Their values were 1214.85 MPa, 1210.83 MPa and 1182.57 MPa (Figure 9a). It also showed the yield stress of the samples, which has the same relationship with the ultimate tensile strength. Compared with the ultimate tensile strength, the yield strength changed more obviously. The size relation of the elongation of samples can be obtained from Figure 9b as follows: SLM-PB-S > SLM-PB-L > SLM-VB. The values were 11.81%, 6.79% and 6.14%, respectively. It is worth noting that from SLM-VB, SLM-PB-L and then to SLM-PB-S, the β phase proportion of the samples gradually increased (Table 5), and the texture of the samples also gradually enhanced (Table 4), which were the main reasons for the enhanced ductility of the SLM samples. Compared with SLM-PB-S, SLM-PB-L has higher porosity, lower relative density and lower grain misorientations (leading to less dislocation accumulation) which are also the main reasons for its lower tensile strength than SLM-PB-S.

### 3.7. Surface Topography

**Figure 10:**
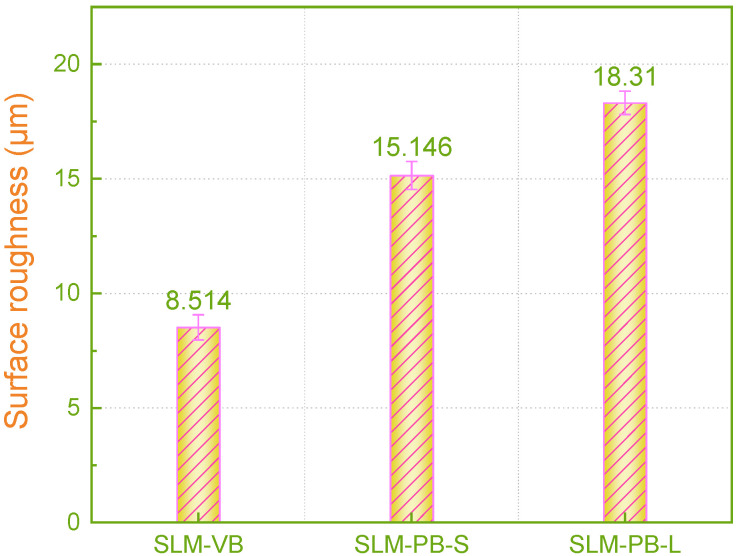
Surface roughness values for SLM Ti6Al4V samples.

**Figure 11:**
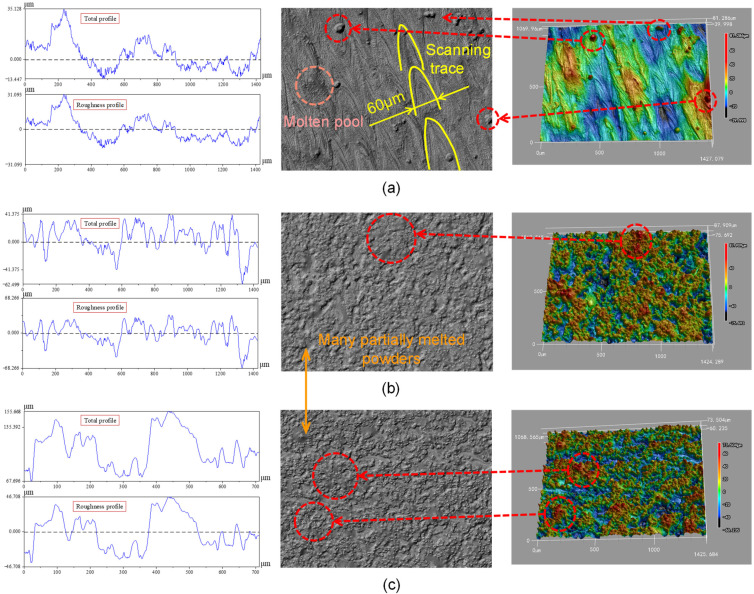
Surface profile, microscopic images and 3D surface analysis of Ti6Al4V fabricated by SLM. (**a**) SLM-VB; (**b**) SLM-PB-S; (**c**) SLM-PB-L.

The evaluation of surface morphology has always been an important indicator of AM samples, because the surface quality of the formed parts directly affects the service performance of the components [40]. Therefore, it is meaningful to analyze the surface quality of the SLM samples fabricated in different building orientations. The test surfaces of SLM-VB, SLM-PB-S and SLM-PB-L were measured and are shown in Figure 10. There are three columns of pictures in Figure 11. The first column on the left shows the curve of roughness value (Ra) of different samples, while the second column shows surface morphology photographed by a laser confocal microscope. The third column shows 3D surface morphology of samples. As shown in the second column of Figure 11a, quantities of laser trajectories were observed in SLM-VB and its width was 60 μm, which was equal to beam spot diameter. There were few unmelted powders that led to smallest surface roughness among the three samples and its value was 8.514 μm. Figure 11b depicts the surface topography of SLM-PB-S, and it shows that its roughness was obviously worse than that of SLM-VB. There were many partially melted powders bonded to the SLM-PB-S surface, which caused rough surface. Its average value was 15.146 μm. The results of Figure 11c showed that in the SLM-PB-L surface condition, there were also a lot of partially melted powders, and its surface roughness was slightly worse than that of SLM-PB-S. In terms of roughness, it was the largest among the three samples whose value was 18.310 μm. After analysis, different building orientations led to different forming methods of SLM samples. SLM-VB was formed by laser scanning, but SLM-PB-S and SLM-PB-L were formed by stacking powder layers. Single scanning forming is more sufficient in powder melting and melt flow than interlayer stack forming. As for the difference in surface quality between SLM-PB-S and SLM-PB-L, the effect of heat accumulation was considered. The heat transfer modes of the SLM-formed surfaces in different building orientations are quite different. The heat transfer modes of the forming process are mainly divided into three types: heat transfers along the formed sample to the substrate, heat transfers along the formed sample to the powder and transfers to the gas environment, as shown in Figure 1. Among the three heat transfer modes, the heat transfer efficiency is the highest along the formed piece to the substrate. Due to the different building orientation of SLM-PB-S and SLM-PB-L, the short side *L*1 of SLM-PB-S was in contact with the substrate compared with the long side *L*2 of SLM-PB-L in contact with the substrate, which reduced the proportion of heat conduction toward the inside of the substrate along the vertical direction. Therefore, it slowed down the heat transfer efficiency during the forming process. As the powder melted more fully, the melt flow was more stable, the spreading was uniform and the surface roughness value was lower.

## 4. Conclusions

In this paper, the influence of building orientation for SLM Ti6Al4V with specific geometry on the microstructure, texture, mechanical and surface properties were studied comprehensively. For horizontal building orientation, the influence of two different contact modes with the substrate on the microstructure, mechanical and surface properties of the SLM samples was studied. In addition, the necessity of limiting the geometry of samples and test surfaces was verified by comparing the study with existing results in the literature. The influence mechanisms of building orientation on multiple factors of microstructure, mechanical and surface properties of SLM Ti6Al4V were clarified. To sum up, the following conclusions are reached.

(1) In the SLM process, different building orientations resulted in different contact areas between the workpiece and the substrate, atmosphere and powder, which affected the heat conduction effect of the forming process. It led to different cooling rates (SLM-VB > SLM-PB-S > SLM-PB-L). The primary β phase was found in SLM-PB-S and SLM-PB-L, and the width of the primary β phase had the following size relationship: SLM-PB-S > SLM-PB-L. All the primary β phases grew along the building orientation.
(2) The grain size, texture strength and two-phase distribution were greatly affected by the building orientation. High cooling rate was conducive to grain refinement and not conducive to the retention of β phase. The sample grain size relationship is as follows: SLM-VB < SLM-PB-S < SLM-PB-L. The texture strength relationship is as follows: SLM-PB-S > SLM-VB > SLM-PB-L. The ratio of β/α is as follows: SLM-PB-S > SLM-PB-L > SLM-VB.
(3) The density, residual stress and surface roughness of the samples were also significantly affected by the building orientation. A high cooling rate led to insufficient melting of powder, resulting in high porosity, which led to a lower density. It also led to uneven plastic deformation, which in turn led to large residual stress. The density relationship of the obtained samples was: SLM-VB > SLM-PB-S > SLM-PB-L. In terms of residual stress, they are all tensile stress, and the size relationship was as follows: SLM-VB > SLM-PB-S > SLM-PB-L. The surface roughness was significantly affected by the forming method, heat transfer type and efficiency, and its value relationship was expressed as: SLM-VB < SLM-PB-S < SLM-PB-L.
(4) With the change in building orientation, the mechanical properties such as microhardness, tensile strength and elongation of the materials also changed greatly. Smaller grain size, stronger texture, larger grain misorientations, smaller porosity and higher density are beneficial to the improvement of mechanical properties. In addition, a high proportion of β phase tends to improve the ductility of the material.
(5) From conclusions (1) to (4), it was found that SLM-PB-S exhibited denser microstructure and better mechanical properties than SLM-PB-L. Therefore, the study on the different modes of horizontal building orientation is significant. The findings of this paper are helpful in selecting proper building orientation in the SLM process. For SLM Ti6Al4V samples, the shorter side in contact with the substrate is beneficial to obtain better mechanical properties when they are fabricated with horizontal building orientation.

## Figures and Tables

**Table 1:** Chemical components of Ti6Al4V powder used in the SLM process.

Elements	Al	V	Fe	O	N	C	H	Ti
Content (Wt. %)	5.5–6.5	3.5–4.5	≤0.25	≤0.13	≤0.03	≤0.08	≤0.012	balance

**Table 2:** The specific parameters in SLM.

Parameter	Value	Unit
Laser power/*P*	145	w
Scan speed/*V*	1000	mm/s
Hatch spacing/*T*	82	μm
Layer thickness/*h*	30	μm
Volume energy density/*E*	58.94	J/mm^3^
Beam spot diameter/*d*	60	μm

**Table 3:** Grain geometry information of SLM Ti6Al4V samples.

Sr. No.	Surface	Mean Area (μm^2^)	Mean Aspect Ratio	Max Aspect Ratio
1	SLM-VB	0.35 ± 0.03	3.49 ± 0.08	18.95 ± 0.56
2	SLM-PB-S	1.08 ± 0.05	3.20 ± 0.07	17.29 ± 0.32
3	SLM-PB-L	2.40 ± 0.06	2.66 ± 0.03	8.43 ± 0.16

**Table 4:** The VFPO and grain sizes of SLM Ti6Al4V samples.

Sr. No.	Surface	VFPO (%)	Average Grain Size (μm)
1	SLM-VB	21.4714 ± 0.0106	0.76 ± 0.07
2	SLM-PB-S	33.1181 ± 0.0235	1.30 ± 0.09
3	SLM-PB-L	20.2455 ± 0.0089	1.95 ± 0.18

**Table 5:** The volume fraction of α phase and β phase.

Sr. No	Surface	Volume Fraction-α (%)	Volume Fraction-β (%)	Volume Ratio (β/α) (%)
1	SLM-VB	99.78 ± 0.04	0.22 ± 0.03	0.222 ± 0.029
2	SLM-PB-S	99.43 ± 0.01	0.57 ± 0.01	0.572 ± 0.011
3	SLM-PB-L	99.46 ± 0.02	0.54 ± 0.01	0.543 ± 0.010

**Table 6:** The residual stress results of the surfaces of different samples.

Sr. No	Test Surface	Residual Stress (MPa)
1	SLM-VB	379.8 ± 89.2
2	SLM-PB-S	246.6 ± 31.6
3	SLM-PB-L	233.3 ± 67.6

## Data Availability

All data are available within the manuscript.

## References

[1] Karolewska K., Ligaj B., Wirwicki M., Szala G. (2020). Strength analysis of Ti6Al4V titanium alloy produced by the use of additive manufacturing method under static load conditions. J. Mater. Res. Technol..

[2] Wu S.Q., Lu Y.J., Gan Y.L., Huang T.T., Zhao C.Q., Lin J.J., Guo S., Lin J.X. (2016). Microstructural evolution and microhardness of a selective-laser-melted Ti-6Al-4V alloy after post heat treatments. J. Alloy. Compd..

[3] Barbieri F.C., Otani C., Lepienski C.M., Urruchi W.I., Maciel H.S., Petraconi G. (2002). Nanoindentation study of Ti6Al4V alloy nitrided by low intensity plasma jet process. Vacuum.

[4] Huaixue L., Baiying H., Fan S., Shuili G. (2013). Microstructure and Tensile Properties of Ti-6Al-4V Alloys Fabricated by Selective Laser Melting. Rare Met. Mater. Eng..

[5] Liu S., Shin Y.C. (2019). Additive manufacturing of Ti6Al4V alloy: A review. Mater. Des..

[6] Wang S., Zhu L., Fuh J.Y.H., Zhang H., Yan W. (2020). Multi-physics modeling and Gaussian process regression analysis of cladding track geometry for direct energy deposition. Opt. Lasers Eng..

[7] Chen Z., Wu X., Tomus D., Davies C.H.J. (2018). Surface roughness of Selective Laser Melted Ti-6Al-4V alloy components. Addit. Manuf..

[8] Cabanettes F., Joubert A., Chardon G., Dumas V., Rech J., Grosjean C., Dimkovski Z. (2018). Topography of as built surfaces generated in metal additive manufacturing: A multi scale analysis from form to roughness. Precis. Eng..

[9] Oyelola O., Crawforth P., M’Saoubi R., Clare A.T. (2018). On the machinability of directed energy deposited Ti6Al4V. Addit. Manuf..

[10] Zhou L., Mehta A., Schulz E., McWilliams B., Cho K., Sohn Y. (2018). Microstructure, precipitates and hardness of selectively laser melted AlSi10Mg alloy before and after heat treatment. Mater. Charact..

[11] Hadadzadeh A., Shalchi Amirkhiz B., Odeshi A., Li J., Mohammadi M. (2019). Role of hierarchical microstructure of additively manufactured AlSi10Mg on dynamic loading behavior. Addit. Manuf..

[12] Kan W.H., Nadot Y., Foley M., Ridosz L., Proust G., Cairney J.M. (2019). Factors that affect the properties of additively-manufactured AlSi10Mg: Porosity versus microstructure. Addit. Manuf..

[13] Li H., Yang Z., Cai D., Jia D., Zhou Y. (2020). Microstructure evolution and mechanical properties of selective laser melted bulk-form titanium matrix nanocomposites with minor B4C additions. Mater. Des..

[14] Han Q., Jiao Y. (2019). Effect of heat treatment and laser surface remelting on AlSi10Mg alloy fabricated by selective laser melting. Int. J. Adv. Manuf. Technol..

[15] Yan X., Yin S., Chen C., Huang C., Bolot R., Lupoi R., Kuang M., Ma W., Coddet C., Liao H. (2018). Effect of heat treatment on the phase transformation and mechanical properties of Ti6Al4V fabricated by selective laser melting. J. Alloy. Compd..

[16] Karami K., Blok A., Weber L., Ahmadi S.M., Petrov R., Nikolic K., Borisov E.V., Leeflang S., Ayas C., Zadpoor A.A. (2020). Continuous and pulsed selective laser melting of Ti6Al4V lattice structures: Effect of post-processing on microstructural anisotropy and fatigue behaviour. Addit. Manuf..

[17] Xie Z., Dai Y., Ou X., Ni S., Song M. (2020). Effects of selective laser melting build orientations on the microstructure and tensile performance of Ti–6Al–4V alloy. Mater. Sci. Eng. A.

[18] Sun D., Gu D., Lin K., Ma J., Chen W., Huang J., Sun X., Chu M. (2019). Selective laser melting of titanium parts: Influence of laser process parameters on macro- and microstructures and tensile property. Powder Technol..

[19] Lee H.Y., Yi S.M., Lee J.H., Lee H.S., Hyun S., Joo Y.C. (2010). Effects of bending fatigue on the electrical resistance in metallic films on flexible substrates. Met. Mater. Int..

[20] Xia M., Gu D., Yu G., Dai D., Chen H., Shi Q. (2016). Influence of hatch spacing on heat and mass transfer, thermodynamics and laser processability during additive manufacturing of Inconel 718 alloy. Int. J. Mach. Tools Manuf..

[21] Qiu C., Panwisawas C., Ward M., Basoalto H.C., Brooks J.W., Attallah M.M. (2015). On the role of melt flow into the surface structure and porosity development during selective laser melting. Acta Mater..

[22] Song J., Wu W., Zhang L., He B., Lu L., Ni X., Long Q., Zhu G. (2018). Role of scanning strategy on residual stress distribution in Ti-6Al-4V alloy prepared by selective laser melting. Optik.

[23] He B., Wu W., Zhang L., Lu L., Yang Q., Long Q., Chang K. (2018). Microstructural characteristic and mechanical property of Ti6Al4V alloy fabricated by selective laser melting. Vacuum.

[24] Ren S., Chen Y., Liu T., Qu X. (2019). Effect of Build Orientation on Mechanical Properties and Microstructure of Ti-6Al-4V Manufactured by Selective Laser Melting. Metall. Mater. Trans. A Phys. Metall. Mater. Sci..

[25] Chang K., Liang E., Huang W., Zhang X., Chen Y., Dong J., Zhang R. (2020). Microstructural feature and mechanical property in different building directions of additive manufactured Ti6Al4V alloy. Mater. Lett..

[26] Hu Z., Zhang H., Zhu H., Xiao Z., Nie X., Zeng X. (2019). Microstructure, mechanical properties and strengthening mechanisms of AlCu5MnCdVA aluminum alloy fabricated by selective laser melting. Mater. Sci. Eng. A.

[27] Liu J., Song Y., Chen C., Wang X., Li H., Zhou C., Wang J., Guo K., Sun J. (2020). Effect of scanning speed on the microstructure and mechanical behavior of 316L stainless steel fabricated by selective laser melting. Mater. Des..

[28] Oyelola O., Crawforth P., M’Saoubi R., Clare A.T. (2018). Machining of functionally graded Ti6Al4V/ WC produced by directed energy deposition. Addit. Manuf..

[29] Balbaa M., Mekhiel S., Elbestawi M., McIsaac J. (2020). On selective laser melting of Inconel 718: Densification, surface roughness, and residual stresses. Mater. Des..

[30] Yang J., Yu H., Yin J., Gao M., Wang Z., Zeng X. (2016). Formation and control of martensite in Ti-6Al-4V alloy produced by selective laser melting. Mater. Des..

[31] Vrancken B., Thijs L., Kruth J.P., Van Humbeeck J. (2012). Heat treatment of Ti6Al4V produced by Selective Laser Melting: Microstructure and mechanical properties. J. Alloy. Compd..

[32] Bai S., Perevoshchikova N., Sha Y., Wu X. (2019). The effects of selective laser melting process parameters on relative density of the AlSi10Mg parts and suitable procedures of the archimedes method. Appl. Sci..

[33] Dong L.X., Wang H.M. (2008). Microstructure and corrosion properties of laser-melted deposited Ti2Ni3Si/NiTi intermetallic alloy. J. Alloy. Compd..

[34] Salmi A., Atzeni E. (2017). History of residual stresses during the production phases of AlSi10Mg parts processed by powder bed additive manufacturing technology. Virtual Phys. Prototyp..

[35] Kuo C.N., Chua C.K., Peng P.C., Chen Y.W., Sing S.L., Huang S., Su Y.L. (2020). Microstructure evolution and mechanical property response via 3D printing parameter development of Al–Sc alloy. Virtual Phys. Prototyp..

[36] Acevedo R., Sedlak P., Kolman R., Fredel M. (2020). Residual stress analysis of additive manufacturing of metallic parts using ultrasonic waves: State of the art review. J. Mater. Res. Technol..

[37] Salmi A., Atzeni E. (2020). Residual stress analysis of thin AlSi10Mg parts produced by Laser Powder Bed Fusion. Virtual Phys. Prototyp..

[38] Bartlett J.L., Li X. (2019). An overview of residual stresses in metal powder bed fusion. Addit. Manuf..

[39] Vishwakarma J., Chattopadhyay K., Santhi Srinivas N.C. (2020). Effect of build orientation on microstructure and tensile behaviour of selectively laser melted M300 maraging steel. Mater. Sci. Eng. A.

[40] Kaynak Y., Tascioglu E. (2020). Post-processing effects on the surface characteristics of Inconel 718 alloy fabricated by selective laser melting additive manufacturing. Prog. Addit. Manuf..

